# Foreign Body Granuloma in the Tongue by a Pequi Spine

**DOI:** 10.1155/2020/8838250

**Published:** 2020-11-10

**Authors:** Rafael Martins Afonso Pereira, Patrícia Cristine de Oliveira Afonso Pereira, Vitor Carvalho Rodrigues, Luiz Fernando Braga de Andrade, Elisa Morais de Carvalho, Helvécio Marangon Júnior

**Affiliations:** School of Dentistry, University Center of Patos de Minas (UNIPAM), Patos de Minas, Minas Gerais, Brazil

## Abstract

Substances considered foreign to the human organism can penetrate it due to local trauma, initially causing an acute inflammatory response against these substances, involving a neutrophilic infiltrate that, when it fails to deal with these foreign bodies, ends up generating a granulomatous inflammatory response. Granuloma formation has been associated with a variety of conditions. The correct clinical and imaging diagnoses are extremely important for the dentist to choose an appropriate therapeutic approach, aiming at the best possible treatment. This work is aimed at describing a case report of a foreign body granuloma, formed on the tongue, from the penetration of a pequi spine, in a 76-year-old patient, in whom, after an imaging diagnosis with ultrasound, surgical removal of the lesion was performed, and the piece was sent for histopathological examination, which confirmed the initial diagnostic hypothesis of a foreign body granuloma. The initial diagnosis of foreign body granulomas is challenging. For this reason, more sophisticated means of diagnosis such as tomography and magnetic resonance become important in the diagnosis, as they can show with greater clarity and reliability the nature of the lesion and its relationship with adjacent anatomical structures. In the case in question, an ultrasound examination was chosen, which was extremely important as an aid to diagnosis, considerably improving surgical planning. In addition, after surgical removal, the result of the histopathological analysis is essential to determine the definitive diagnosis, as it determines the granulomatous characteristic of the lesion.

## 1. Introduction

True granulomas are compact microscopic structures, formed by the collection of epithelioid histiocytes, surrounded by inflammatory cells and filled with fibroblasts and collagen fibers, in response to the persistence of some stimulus [1–3]. The presence of multinucleated giant cells, formed by the coalescence of macrophages, can also be observed [2].

A foreign body granuloma can develop from several endogenous or exogenous substances [4]. Endogenous substances, such as cholesterol from cell membranes, keratin, and hair, can originate foreign body granulomas. The introduction of foreign bodies in oral and peribuccal tissues is related to traumatic events, dental procedures, and cosmetic dermatological procedures [5–7]. Amalgam, suture threads, endodontic sealants, and gutta-percha are some examples of dental materials capable of causing a foreign body granulomatous reaction [4].

Granulomatous inflammation in the hard and soft structures of the mouth is an uncommon event [2]. Furthermore, to date, there are no reports in the literature of a case involving the spine of the pequi fruit (*Caryocar brasiliense*) as an etiological factor of the lesion. Thus, the present study is aimed at reporting an unprecedented case of a foreign body granuloma in the tongue caused by pequi spines.

## 2. Case Report

**Figure 1:**
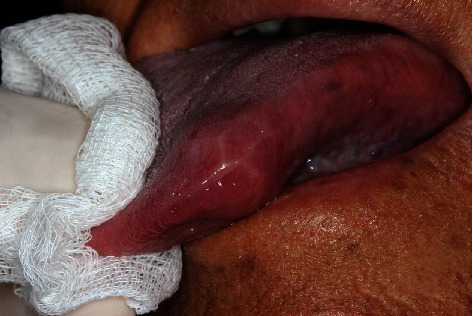
Presence of a free, well-defined, rounded nodule, with the tongue showing normal color and an enlarged aspect in the central region on visual clinical examination.

A 76-year-old male patient sought a maxillofacial surgeon because of difficulty in swallowing and in chewing, pain complaint, and an internal nodule hardened on the tongue. On visual and palpatory clinical examination, a free and well-defined rounded nodule was found, with the tongue showing normal color, but with an enlarged aspect in the central region (Figure 1). In the initial clinical interview, the patient did not remember whether he had eaten anything that had hurt his tongue. To assist in the diagnosis and try to elucidate the fact, the patient was asked for a complementary ultrasound examination of the tongue. The ultrasound device was a linear matrix probe, with a frequency of 12 Mhz. In the result, it was possible to observe a 5 mm hyperechoic linear image with a 1.2 × 0.7cm granuloma formation. The lesion was located on the left lateral border of the distal third of the patient's tongue, 0.1 cm from the surface.

**Figure 2:**
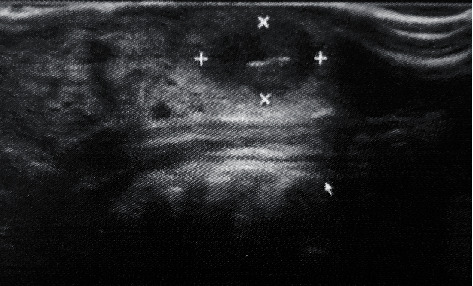
Ultrasonography showing a circumscribed nodular lesion. The echographic aspect was suggestive of a foreign body inside, suggesting an image compatible with a thorn, with granuloma formation.

**Figure 3:**
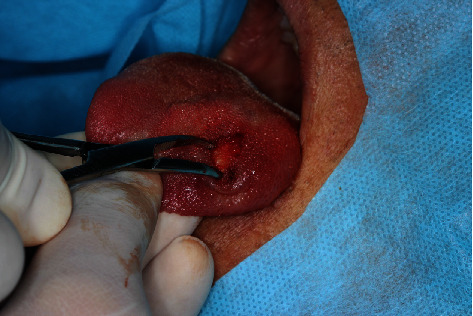
Surgical access performed through a linear incision in the lingual dorsum on the left side, close to the most superficial region of the lesion.

**Figure 4:**
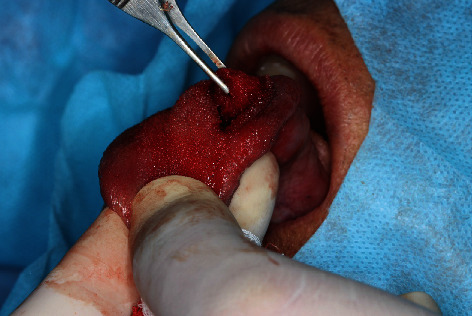
Divulsion of the lesion, using blunt-tip instruments, from the adjacent tissue planes, having been cleaved without the rupture of its evident lining capsule.

**Figure 5:**
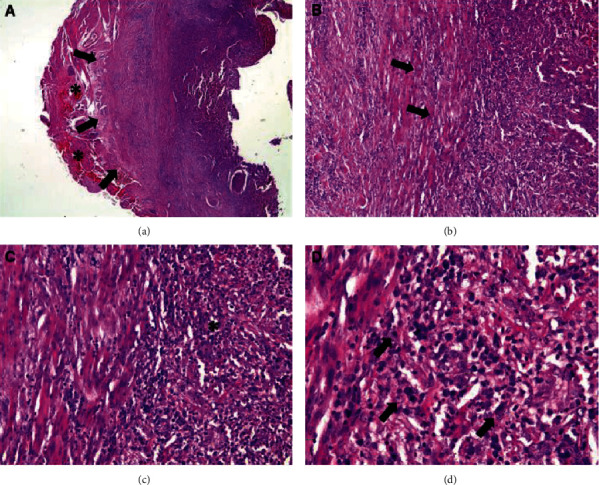
Histopathological examination showing a fragment of a fibrous tissue nodule well delimited by means of a capsule, with thick collagen fibers richly peripherally vascularized and with sparse chronic inflammatory cells, in addition to numerous macrophages that stand out in the midst of immune-inflammatory cells: (a) 50x magnification: the full black arrows represent the outer limit of the fibrous capsule, and the black asterisks represent the peripheral vascular congestion; (b) 100x magnification: the full black arrows represent the bundles of collagen fibers; (c) 200x magnification: the black asterisk represents the chronic inflammatory infiltrate; (d) 400x magnification: the full black arrows represent numerous macrophages in the middle of the chronic inflammatory infiltrate.

**Figure 6:**
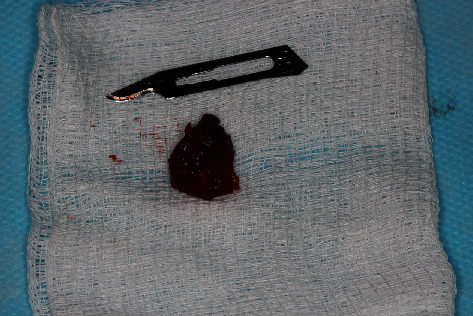
Total enucleation without compromising the capsule.

The echographic aspect suggested a foreign body inside, showing an image compatible with a spine, with granuloma formation (Figure 2). On the patient's return, when asked about the possible spine on the tongue, he recalled that he had eaten pequi a while ago but that he was not sure exactly regarding the time, leading us to a more consistent diagnostic hypothesis of a foreign body granuloma caused by a pequi spine. Complete surgical enucleation of the lesion was then performed, with bilateral anesthetic block of the lingual nerve. Surgical access was performed through a linear incision in the lingual dorsum on the left side, close to the most superficial region of the lesion (Figure 3). The lesion was divulsed, using blunt-tipped instruments, from the adjacent tissue planes, having been cleaved without the rupture of its evident lining capsule (Figure 4). No vascular rupture with subsequent hemorrhagic accident occurred, despite the close proximity of the lesion with lingual arteries and veins. The visualization of these large lingual vessels was perceived by the surgeon during the operation. After total enucleation without compromising the capsule (Figure 5), the synthesis was performed both in deep muscle planes and on the epithelial surface using simple isolated points. The surgical specimen removed by excisional biopsy was sent for anatomopathological examination, which confirmed the initial diagnostic hypothesis of a foreign body granuloma from the penetration of a pequi spine. Histopathological examination revealed a nodule of fibrous tissue well delimited by means of a capsule, with thick collagen fibers richly peripherally vascularized and with sparse chronic inflammatory cells, in addition to numerous macrophages that stand out in the midst of immunoinflammatory cells (Figure 6).

## 3. Discussion

There are voluntary and involuntary reasons for foreign substances to penetrate people. Materials for tattoos and aesthetic fillings are included in the first group, while the second is related to substances capable of causing cutaneous trauma and their consequent inclusion in tissues [8], such as glass fragments [9] and bee sting [10]. In the present case, the penetration of the foreign body, pequi spine, happened involuntarily, when the patient was eating. The pequi is a fruit composed of several layers, among them the prickly endocarp, which has the function of protecting the edible seed [11], and, for this reason, it is necessary for people to be cautious during their chewing.

A vegetable foreign body granuloma still has an uncertain etiopathogenesis. However, what is more accepted in the literature is that granulomatous lesions are the result of implantation of foreign bodies of plant origin, which have in their composition an amorphous eosinophilic material surrounded by a hyaline ring [12]. After the foreign body penetrates the tissues, the starch, which constitutes the amorphous material, is rapidly metabolized and eliminated by the human organism, while the hyaline ring, composed of cellulose, remains intact and produces an inflammatory response in the organism [12, 13]. It is common that in these cases, the foreign body of plant origin is associated with other diseases, being of fundamental importance to identify the initial cause of the pathological process [14]. In the present case, as the foreign body granuloma was isolated, that is, being the primary disease, the simple removal of the irritant (pequi spine) was enough for the treatment to be successful.

The time for the appearance of a foreign body granuloma can vary from a few days to years [15], and, clinically, it presents as a local inflammatory reaction, associated or not with purulent secretion, pain [3, 5, 16], material migration [5, 16], slight swelling, ulcerations [4], and color changes, which range from grayish black to bright red [17]. It is rare to find cases in which the foreign body granuloma developed in the oral cavity is asymptomatic [3, 5, 16]. The signs and symptoms found in the case in question confirm what is described in the literature, since the patient had noticeable swelling on the side of the tongue, pain, and difficulty in swallowing.

A foreign body granuloma presents a diagnostic challenge to the dentist [18] and, therefore, requires the association of data collected, during the interview, clinical findings, and complementary imaging tests [16], since these reproduce important characteristics for the diagnosis [19]. The visualization of foreign bodies is related to both the density and the proximity of the tissues, as well as the location in which they are inserted [18]. For the location of foreign bodies in soft tissues, magnetic resonance imaging is a good alternative [18]; however, ultrasonography has proved to be quite effective in identifying foreign body granulomas [20]. To establish the definitive diagnosis, it is necessary to perform a histopathological examination, considered the gold standard technique [16, 21]. In the present case, during the clinical interview, the patient did not present any systemic or local evidence that could explain the genesis of the lingual lesion perceived on physical examination. After the ultrasound result and in view of the ultrasound findings that demonstrated a hyperechoic image at the center of a region compatible with a granulomatous reaction, the hypothesis of a foreign body granuloma was raised, being reinforced, at this moment, by the patient's previous recall that, during his feeding and ingestion of the pequi, an accident had occurred and that inadvertently a spine, contained in the center of the fruit, had pierced his tongue which could have been introduced in a deep tissue plane.

Granulomatous inflammations have different etiopathogenesis; however, they show similar histopathological patterns [17] and great clinical similarities. It is necessary to establish possible differential diagnoses: mucoceles, neoplasms of mesenchymal soft tissues, orofacial granulomatosis, angioedema, and Melkersson-Rosenthal syndrome [5, 22]. In this clinical case, the ultrasound result was very presumptive, demonstrating, therefore, the importance of this complementary imaging exam in helping to clarify the diagnosis in dentistry.

Aesthetic procedures, in an attempt to slow down aging through injectable soft tissue fillers, are increasingly frequent [23]. However, the increasing performance of these procedures, regardless of the type of material used, also causes an increase in the number of side effects [24], the most common being foreign body granulomas [25, 26]. The treatment approach for this type of granulomatous reaction by an exogenous foreign body has good resolvability through intralesional injections of corticosteroids [21, 26]. On the other hand, when foreign body granuloma lesions are caused by other exogenous factors, such as that presented by this clinical case, surgical excision by enucleation of the lesion should be the first option of choice, since this technique can be used both for definitive laboratory diagnosis and for therapy [7, 26, 27].

The surgical treatment adopted, aided by a thorough diagnosis by means of an appropriate complementary imaging exam, selected for the case, allowed for a conservative surgical approach, with a comfortable immediate postoperative period, good recovery, and efficient long-term tissue repair with excellent prognosis, once the cause of the granulomatous inflammatory reaction has been removed.
